# A zebrafish model for studying the mechanisms of newborn hyperbilirubinemia and bilirubin-induced neurological damage

**DOI:** 10.3389/fcell.2023.1275414

**Published:** 2023-11-14

**Authors:** Metehan Guzelkaya, Ebru Onal, Emine Gelinci, Abdullah Kumral, Gulcin Cakan-Akdogan

**Affiliations:** ^1^ Izmir Biomedicine and Genome Center, Izmir, Turkiye; ^2^ Institute of Health Sciences, Dokuz Eylül University, Izmir, Turkiye; ^3^ Division of Neonatology, Department of Pediatrics, Faculty of Medicine, Dokuz Eylul University, Izmir, Turkiye; ^4^ Department of Medical Biology, Faculty of Medicine, Dokuz Eylül University, Izmir, Turkiye

**Keywords:** hyperbilirubinemia, zebrafish, newborn, BIND, neurotoxicity

## Abstract

Unresolved neonatal hyperbilirubinemia may lead to the accumulation of excess bilirubin in the body, and bilirubin in neural tissues may induce toxicity. Bilirubin-induced neurological damage (BIND) can result in acute or chronic bilirubin encephalopathy, causing temporary or lasting neurological dysfunction or severe damage resulting in infant death. Although serum bilirubin levels are used as an indication of severity, known and unknown individual differences affect the severity of the symptoms. The mechanisms of BIND are not yet fully understood. Here, a zebrafish newborn hyperbilirubinemia model is developed and characterized. Direct exposure to excess bilirubin induced dose- and time-dependent toxicity linked to the accumulation of bilirubin in the body and brain. Introduced bilirubin was processed by the liver, which increased the tolerance of larvae. BIND in larvae was demonstrated by morphometric measurements, histopathological analyses and functional tests. The larvae that survived hyperbilirubinemia displayed mild or severe morphologies associated with defects in eye movements, body posture and swimming problems. Interestingly, a plethora of mild to severe clinical symptoms were reproduced in the zebrafish model.

## 1 Introduction 

Bilirubin is the end product of hemoglobin breakdown, a beneficial molecule at low levels due to its antioxidant properties, but becomes toxic when elevated (Fujiwara et al., 2018). Unconjugated bilirubin (UCB) is carried by serum albumin protein to the liver, where it is conjugated to glucuronic acid by hepatic UDP glucuronosyltransferase 1A1 (UGT1A1), and the produced bilirubin-glucronidic acid conjugates are secreted into bile. Increased erythrocyte turnover leads to increased bilirubin production, and the delayed activation of UGT1A1 in newborns can lead to hyperbilirubinemia (Ullah et al., 2016; Burchell et al., 2017). Jaundice is the mild form of neonatal hyperbilirubinemia that occurs in 60% of term neonates and almost all preterm newborns and often self-resolves within days (Ullah et al., 2016). However, infants may develop severe hyperbilirubinemia when serum bilirubin levels increase above 20 mg/dL, and UCB can accumulate in tissues including the brain (Olusanya et al., 2018). UCB accumulation in the central nervous system can lead to bilirubin-induced neurological damage (BIND) (Maisels and Kring, 2006). To prevent BIND, infants with significant hyperbilirubinemia are first treated with phototherapy, which increases the amount of soluble bilirubin photo isomers. Next, exchange transfusion is performed to eliminate excess bilirubin from the serum (Murki and Kumar, 2011; Mreihil et al., 2015). BIND may result in acute bilirubin encephalopathy (ABE) or chronic bilirubin encephalopathy (CBE, also called kernicterus spectrum disorder (KSD)) that leaves permanent neurological damage characterized by oculomotor problems, auditory neuropathy, muscle tonus and movement anomalies (Pranty et al., 2022). Individual factors such as serum albumin levels and bilirubin binding capacity, genetic susceptibilities, metabolic differences, factors that control central nervous system exposure to bilirubin and bilirubin clearance from the CNS are thought to affect the progression of the disorder (Watchko and Tiribelli, 2013).

Although some animal models have been developed, the molecular mechanisms of BIND in infants and the predisposing factors are not fully understood. The *ugt1* mutant rat (Gunn Rat) is a genetic model with lifelong hyperbilirubinemia and a good model to simulate Criegler-Najjar syndrome (Bortolussi and Muro, 2020). Another genetic model is generated in mice, which induces neonatal lethality (Bortolussi et al., 2012). Induction of hemolysis by drug administration or direct injection of bilirubin into the brain are among the methods to induce newborn hyperbilirubinemia (Rice and Shapiro, 2008; Song et al., 2014; Amini et al., 2017). Each model has its own advantages and disadvantages, as well as technical challenges, and the need for *in vivo* models that will allow mechanistic studies to be performed to understand BIND mechanisms and develop preventive strategies is not fulfilled (Amini et al., 2021; Pranty et al., 2022).

The zebrafish model can offer advantages such as ease of live imaging throughout embryonic and larval stages, ease of chemical exposure and genetic modifications. However, no zebrafish hyperbilirubinemia model has been developed to date, although bilirubin metabolism seems to be conserved in zebrafish. Zebrafish produce hemoglobin as of 48 h post fertilization (hpf), and *heme oxygenase* and *biliverdin reductase* genes are expressed at this stage (Holowiecki et al., 2016). The transition from embryo to free-feeding larvae is completed by 120 hpf, while the gastrointestinal system and liver gain full functionality, and liver UGT enzymes are expressed (Kimmel et al., 1995; Field et al., 2003; Chu and Sadler, 2009; Wang et al., 2014). Based on this knowledge, we hypothesized that the newly hatched larvae at 48 hpf resemble a newborn baby in terms of bilirubin metabolism and liver function and that the window for neonatal hyperbilirubinemia induction should be between 2 and 5 dpf. Accordingly, hyperbilirubinemia was induced by direct exposure of early larvae to bilirubin. The conditions of acute hyperbilirubinemia induction, tissues of bilirubin accumulation, routes of bilirubin elimination, and histopathology of diseased larvae were examined. Finally, the lasting neurological effects of acute hyperbilirubinemia were examined in recovered larvae. Several clinical symptoms were successfully reproduced in the zebrafish neonatal hyperbilirubinemia model.

## 2 Materials and methods

### Zebrafish maintenance

Zebrafish were reared under standard conditions at 28°C under a 14/10 h light/dark cycle at the Izmir Biomedicine and Genome (IBG) Center Zebrafish Vivarium. The Casper zebrafish line (roy^−/−^; nacre^−/−^) embryos were incubated at 28°C in E3 (5 mM NaCl, 0.17 mM KCl, 0.33 mM CaCl_2_·2H_2_O and 0.33 mM MgCl_2_·6H_2_O, 1% methylene blue) medium before and during the experiment (White et al., 2008).

### Bilirubin treatment

85.5 mM bilirubin stock was prepared in 0.25 M NaOH, 6 mM bovine serum albumin (BSA) stock was prepared in PBS. Bilirubin:BSA (3:1) solution containing 900 µM bilirubin was prepared by diluting BSA in PBS and adding the required volume of 85.5 mM bilirubin. This intermediate stock was aliquoted and frozen, and fresh dilutions were made in E3 at the time of treatment. Dechorionated 48 hpf larvae were treated in 24-well plates, protected from light and incubated at 28°C. At the end of exposure, larvae were washed with E3 three times to remove excess bilirubin.

### Imaging and quantitation

Larvae were imaged live (anesthetized with tricaine) or after fixation with 4% formaldehyde. Samples were embedded in 1% low melting agarose and mounted on glass bottom petri dishes. An Olympus SZX10 stereomicroscope and Zeiss LSM880 confocal microscope were used for imaging. The sizes of larval structures and the relative amount of accumulated bilirubin were measured with ImageJ software using brightfield stereomicroscopy to record dorsal images of the larvae. For bilirubin quantitation, the RGB images were converted to grayscale with ImageJ (version 1.53q), and inverted images were used to measure the mean gray value (MGV) in defined areas.

### Spectrophotometric analysis

Sixty larvae were crushed in 80 μL of PBS, and debris was removed by centrifugation at 6000 rpm for 4 min. Twenty microliters of 0.25 M NaOH solution was gently pipetted onto the supernatants to solubilize bilirubin. The mixture was centrifuged for 2 min at 6000 rpm. Seventy microliters of supernatant was transferred to 96-well plates, and the absorbance spectrum was recorded at 400–500 nm using a spectrophotometer. The peak absorbance was found to be at 420 nm. A bilirubin standard curve was obtained by measuring spectra of 0.01, 0.03, 0.05, 0.1, 0.2, 0.3, 0.4, 0.5, and 0.8 mg/dL bilirubin:BSA in control larval lysates. The formula (y = −6.1064 × ^2^ + 8.1667x–0.0095; R^2^:0.99) obtained from the concentration/absorbance plot was used to calculate the bilirubin concentration in the lysates. The amount of average bilirubin retained in the larval body was reported as ng.

### Gaze limitation test

6 dpf zebrafish were placed in 3% methylcellulose without anesthesia, 30-second-long videos were recorded dorsally under a stereomicroscope, and light flashes were applied 5 times with 6-s intervals. Turn angles were calculated with ImageJ.

### Quantitation of free swimming

Larvae were imaged in 24-well plates at 4 dpf and 6 dpf with a stereomicroscope. Twenty-five frames per second and 3-minute-long videos were recorded with an SC50 camera. Trace lengths and speeds were analyzed using the Tracker 6.1.3 application auto tracker tool from Open-Source Physics (OSP).

### Statistical analyses

Statistical significance analysis was performed with GraphPad Prism software. Student’s t-test was used for comparison of two groups, and one-way ANOVA was used for comparison of several groups to one control.

### Histopathological analysis

Six dpf zebrafish larvae were fixed with 10% neutral buffered formalin overnight at 4°C and processed through a series of alcohol and xylene to embed in paraffin (Sabaliauskas et al., 2006). One micrometer sections were obtained with a microtome. Hematoxylin and eosin-stained sections were imaged (Olympus BX61).

## 3 Results

### 3.1 Phenotypes of bilirubin-exposed larvae

**FIGURE 1:**
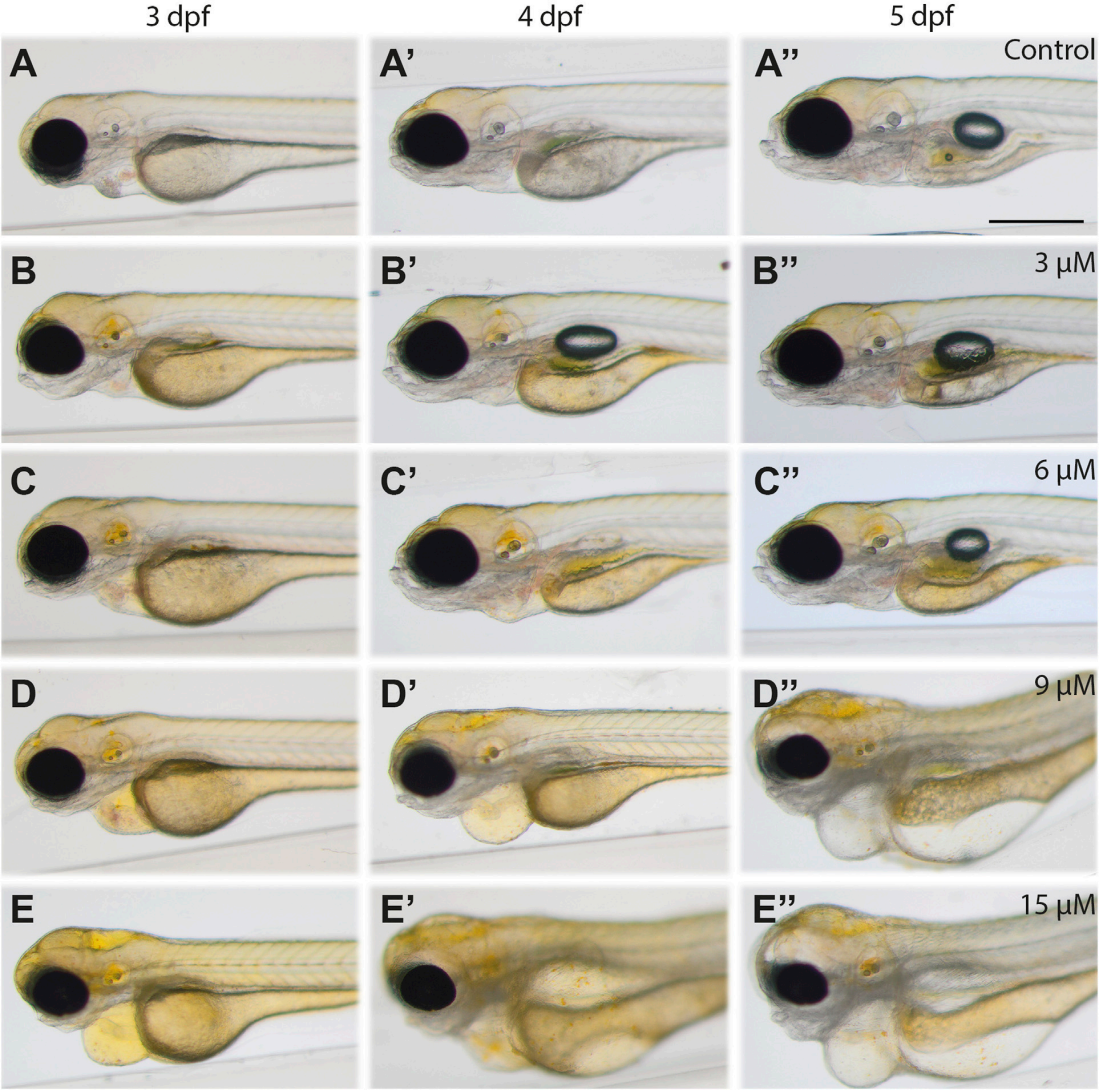
**Images of bilirubin-exposed larvae:** Larvae were exposed to **(A-A′′)** carrier, **(B-B′′)** 3 μM, **(C-C′′)** 6 μM, **(D-D′′)** 9 μM, and **(E‒E′′)** 15 µM bilirubin between 2–3 dpf. Representative images recorded at the end of treatment (3 dpf, left column), 1 day after washout (4 dpf, middle column) and 2 days after washout at 5 dpf (right column) are displayed in the figure. Scale bar: 200 μm, n = 40.

To induce hyperbilirubinemia in zebrafish, bilirubin ranging from 3 μM to 45 µM was added to the aqueous medium, and the zebrafish larvae were treated for 24 h or 48 h, starting from 48 hpf. The effects of excess bilirubin on the developing zebrafish were monitored by imaging of the larvae every 24 h until 5 dpf (Figure 1 and Supplementary Figure S1). The yellow color of bilirubin, which penetrated the zebrafish body, was easily detected with brightfield stereomicroscope imaging.

Upon 24 h of exposure between 2–3 dpf, 3 µM or 6 µM bilirubin caused yellow coloration of the head and accumulation in the otic vesicle but did not induce any morphological defects or toxicity. After washout, gradual elimination of bilirubin from the body was observed at 4 dpf and 5 dpf. While the brain became less yellow, the gut accumulated more bilirubin. On the other hand, exposure to 9 μM and 15 µM bilirubin caused more prominent coloration of the whole body, with significant bilirubin deposition in the brain, otic vesicles and other tissues. Pericardial edema was observed at the end of 24 h of exposure (Figures 1D,F).

Treatment with 30 µM bilirubin caused lethality with full penetrance. One day after the washout, at 3 dpf, some bilirubin was detected in the gut area; however, the body was still yellow (Figures 1D′, F′). The toxicity of 9–15 µM bilirubin was permanent; the treated larvae had heart and yolk edema, darkening of the brain and muscle structures at 4 dpf (Figures 1D′, F′), and major deterioration of the larval body with smaller head, damaged gut, yolk edema and curved body was observed at 5 dpf (Figures 1D″, F″).

**FIGURE 2:**
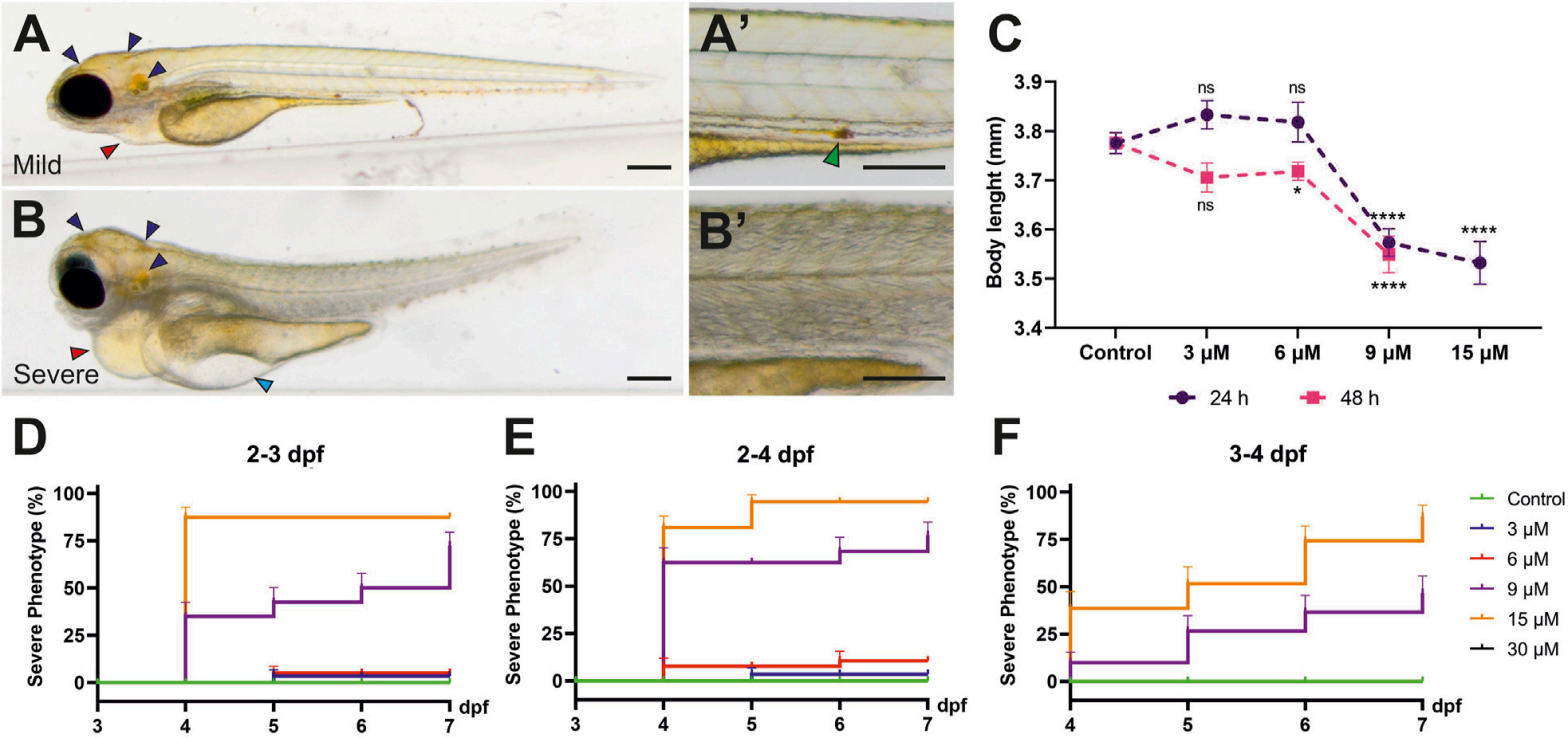
**Mild and severe phenotypes:** The larvae exposed to 9 µM bilirubin between 2–3 dpf display **(A)** mild or **(B)** severe phenotypes at 4 dpf. Close-up images are shown next to the overview images. navy, blue and red arrows represent bilirubin, yolk edema and pericardial edema, respectively. The green arrow indicates bilirubin excreted from the GI tract. Scale bars: 200 µm. **(C)** Average body length of larvae treated with bilirubin at 2–3 dpf for 24 h (purple line) or at 2–4 dpf for 48 h (pink line) shows a significant shortening of body length upon 9–15 µM bilirubin exposure. **(D–F)** Penetrance of the severe phenotype after exposure to bilirubin between **(D)** 2–3 dpf, **(E)** 2–4 dpf and **(F)** 3 - 4 dpf. Larvae were treated with vehicle, 3 μM, 6 μM, 9 µM or 15 µM bilirubin. The mean ± SEM was calculated from triplicate treatments of 15 larvae in each group.

Individual differences were observed among the treated larvae. While some larvae had a mild phenotype with heart edema, others displayed a severe phenotype with swelling around the eyes and in the head, major pericardial and yolk sac edema, distorted body posture, deteriorated muscles and loss of transparency (Figures 2A,B). Toxicity induced by 9–15 µM bilirubin exposure for 24 h led to a significant shortening of the larval body, as indicated by total length quantification of all treated larvae at 4 dpf (Figure 2C). Tested lower doses did not affect the body length upon 24 h treatment, while 48 h treatment with 6 µM bilirubin caused a mild but significant shortening of the body (Figure 2C). Penetrance of the severe phenotype increased with a higher bilirubin dose. Thirty-four percent of larvae treated with 9 µM bilirubin between 2 and 3 dpf displayed a severe phenotype, whereas 15 µM bilirubin induced a severe phenotype in nearly 90% of the larvae (Figure 2D). From 4 to 7 dpf, progression of existing symptoms and an increase in the prevalence of a severe phenotype were observed in all groups. An increase in exposure time to 48 h did not cause any toxicity at 3 or 6 µM doses, although a minor build-up of bilirubin in the body was observed (Figure 2E, Supplementary Figure S2). However, 9 µM bilirubin treatment for 48 h induced a severe phenotype in 60% of the treated larvae, and 15 µM led to a severe phenotype, which caused lethality in 90% of larvae by 5 dpf (Figure 2E). Overall, these data showed that the accumulation of bilirubin in zebrafish tissues and the damage induced by hyperbilirubinemia increased in a dose- and exposure time-dependent manner. Since clearance of bilirubin relies on liver, kidney and gut function, we hypothesized that if bilirubin exposure starts later during development, bilirubin clearance would be more efficient, resulting in less toxicity (Thomas et al., 2021). As expected, when bilirubin exposure was applied between 3–4 dpf, the larvae tolerated higher doses of bilirubin, and the incidence of a severe phenotype was reduced (Figure 2F).

### 3.2 Bilirubin is processed by the liver and gut in zebrafish larvae

**FIGURE 3:**
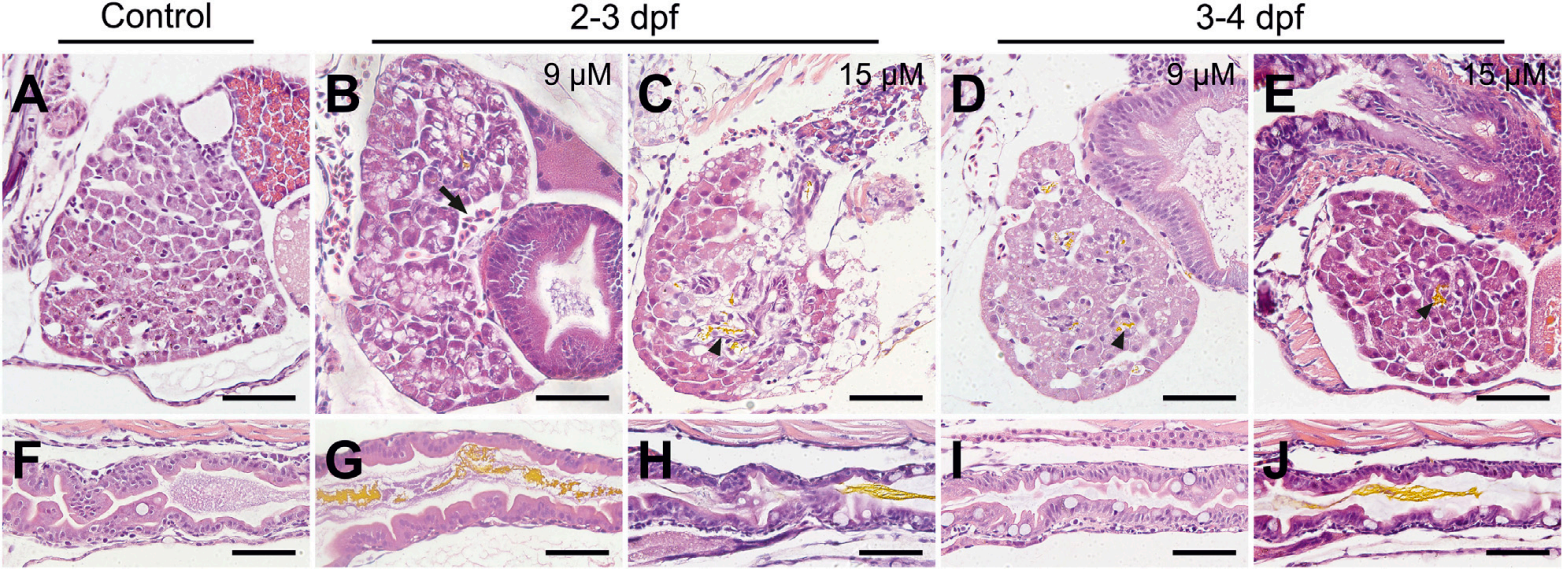
**Histopathology of the liver and gut after bilirubin exposure.** **(A–E)** Liver and **(F–J)** gut tissues of 6 dpf larvae are stained with H & E after paraffin sectioning. **(A and F)** Control larvae treated with vehicle. Larvae exposed to **(B and G)** 9 µM and **(C and H)** 15 µM bilirubin between 2 and 3 dpf. Larvae exposed to **(D and I)** 9 μM, **(E and J)** 15 µM bilirubin between 3 - 4 dpf. Arrow: enlarged vein, arrowhead: bilirubin in bile duct, scale bars: 50 µm.

Histopathological analysis was performed to visualize bilirubin in tissues and investigate the effects of bilirubin on the liver and gut, which are organs involved in bilirubin metabolism and excretion. Since the development of the gastrointestinal system and liver is completed at the end of 5 dpf, sections were taken on 6 dpf larvae that were exposed to 9 or 15 µM bilirubin between 2–3 dpf or 3–4 dpf. Liver damage was visible in larvae that received bilirubin between 2–3 dpf (Figures 3A–C). Liver vacuolation, disruption of hepatocyte tubules and enlargement of hepatic vessels were detected in the 9 µM group (Figure 3B). Liver degeneration was more severe in the 15 µM group, where large clusters of bilirubin were detected in disturbed bile ducts, large vacuoles, breakdown of hepatocytes, and hepatocytes with enlarged or small nuclei were observed (Figure 3C). The larvae that received 9 µM bilirubin treatment between 3–4 dpf had healthy livers with normal hepatocytes, hepatocyte tubules and general morphology (Figure 3D). Detection of bilirubin in the liver also supports the hypothesis that bilirubin is metabolized in zebrafish larval liver and excreted into the bile. Although 15 µM bilirubin exposure between 3–4 dpf resulted in some toxicity, the livers of these larvae were healthier (Figure 3E). Hepatocytic vacuoles were not observed; however, the cytoplasm of the hepatocytes was basophilic, as in the 9 µM 2–3 dpf treated group (Figures 3B,E).

Damage to the gut was evident in 2–3 dpf exposed larvae, such as disorganization of enterocytes, loss of gut folds and disruption of the mucosal layer when compared to the control (Figures 3F–H). Moreover, an increase in goblet cells was apparent. Later exposure to bilirubin was better tolerated in the gut, especially at the 9 µM dose, and the gut structure was not significantly affected. No bilirubin was detected in the lumen, although an increase in goblet cell number was observed in some larvae (Figure 3I). Finally, exposure to 15 µM bilirubin between 3 and 4 dpf affected the gut structure to the extent that folds were disturbed and some enterocytes were disorganized (Figure 3J).

### 3.3 Bilirubin accumulation in the larval body and brain

**FIGURE 4:**
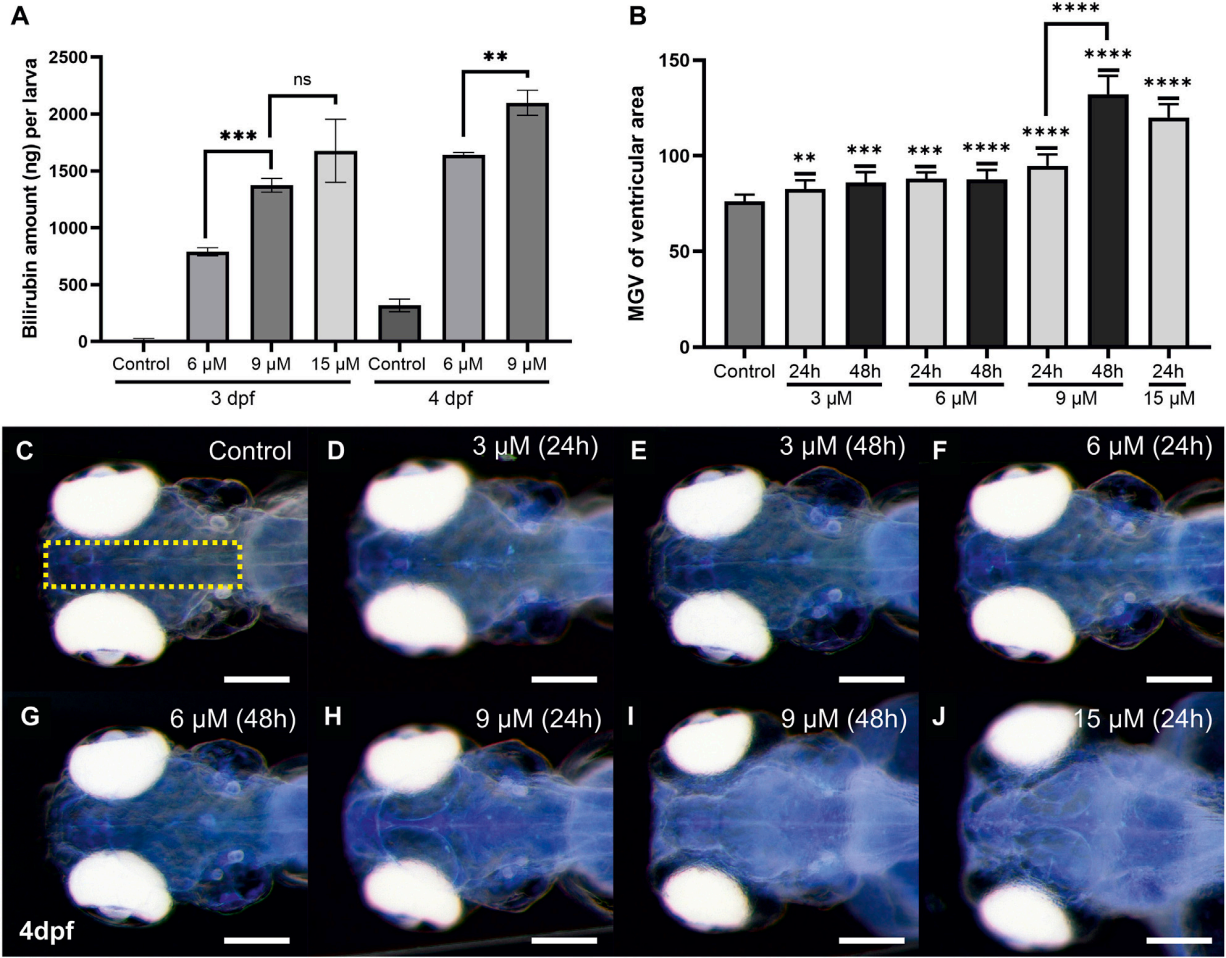
Quantitative analyses of bilirubin accumulation: **(A)** Bilirubin concentration in larval body was measured from lysates, after exposure to 6 and 9 µM bilirubin for 24 or 48 h, mean values were plotted (n = 60 for each group, experiment was done in triplicate). **(B)** Relative bilirubin accumulation in the brain at 4 dpf was plotted for each group (n = 15). **(C–J)** Inverted images of the head that show bilirubin accumulation as bright blue spots were used for the graph in **(B)**. Scale bar: 200 µm.

Total bilirubin retained in the body of the treated larvae was quantified spectrophotometrically. To this end, larval lysates were obtained, bilirubin was solubilized by the addition of NaOH to the suspension, and bilirubin absorbance was measured. The amount of retained bilirubin in the larval body was calculated in each experimental condition. Larvae exposed to 6 µM bilirubin for 24 h (2–3 dpf) accumulated approximately 780 ng of bilirubin in their body (Figure 4A). Exposure to 9 μM and 15 µM bilirubin led to 1,373 ng and 1,676 ng bilirubin retention in the larval body, respectively. 48-h exposure to low dose of 6 µM induced 1,643 ng bilirubin accumulation, whereas maximum measured bilirubin accumulation, 2,099 ng, was found in larvae treated with 9 µM bilirubin for 48 h.

The spatial distribution of bilirubin depositions in the brain was observed easily when larval heads were imaged dorsally with a stereomicroscope at 4 dpf (Supplementary Figure S3). The depositions were most concentrated in the ventricles and spread to a wider area as the dose and exposure time increased (Supplementary Figure S3). Bilirubin (9 or 15 µM) led to increased bilirubin staining throughout the brain, making the telencephalon and rhombencephalon readily distinguishable (Supplementary Figures S3F–H). Accumulation of bilirubin in the brain was quantified by measuring the bilirubin signal at the middle of the brain images, and average signal intensities were plotted (Figures 4B–J). A dose dependency was observed, and maximum accumulation was detected after 24 h of exposure to 15 µM. Exposure to 3 or 6 µM bilirubin for 48 h did not further increase the accumulation. On the other hand, 9 µM bilirubin exposure resulted in increased accumulation at 48 h (Figures 4B–J). Quantitation of bilirubin intensity in the brain was also performed at 3 dpf and 5 dpf, and a similar trend was detected (Supplementary Figure S4). Similarly, bilirubin accumulation in the otic vesicle increased in a dose- and time-dependent manner (Supplementary Figure S5).

### 3.4 Hyperbilirubinemia caused neural damage in zebrafish larvae

**FIGURE 5:**
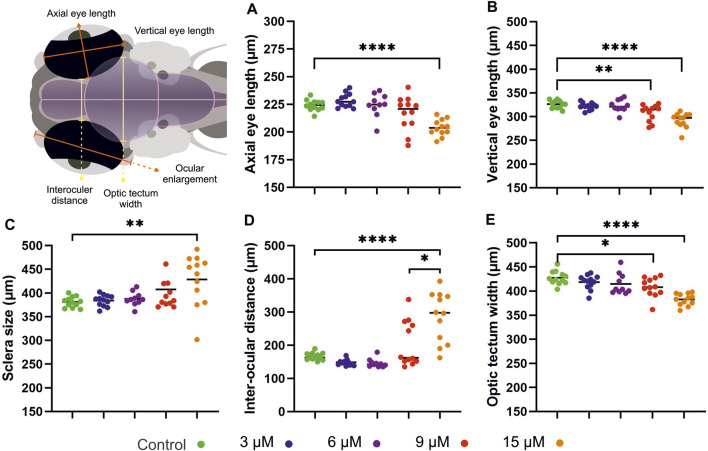
**Morphologic analysis of neural tissues in bilirubin-exposed larvae:** Larvae exposed to 3 μM, 6 μM, 9 μM, and 15 µM bilirubin between 2 and 3 dpf were imaged at 4 dpf. **(A)** Axial eye length and **(B)** vertical eye length decreased; **(C)** sclera size and **(D)** interocular distance increased; **(E)** optic tectum width decreased upon hyperbilirubinemia induction. (n = 24, n = 12, n = 10, n = 19 and n = 13 for control group, 3 μM, 6 μM, 9 μM and 15 µM).

Hyperbilirubinemia induced the development of edema in the brain and around the eyes by 4 dpf, coupled with shrinking of these tissues (Supplementary Figures S3G, H). Eye size decreased, and the sclera of the ocular tissue was enlarged in a dose-dependent manner (Figure 5). Significant reductions in vertical and axial eye lengths were observed in both the 9 μM and 15 µM bilirubin-treated groups. Sclera enlargement was more prominent in the 15 µM-treated group, with a 20% increase in size. This enlargement was also observed at the back of the eyeball, which caused an increase in the interocular distance. On the other hand, brain tissue appeared smaller, and a significant dose-dependent reduction in the optic tectum was observed (Figure 5E, Supplementary Figure S3).

**FIGURE 6:**
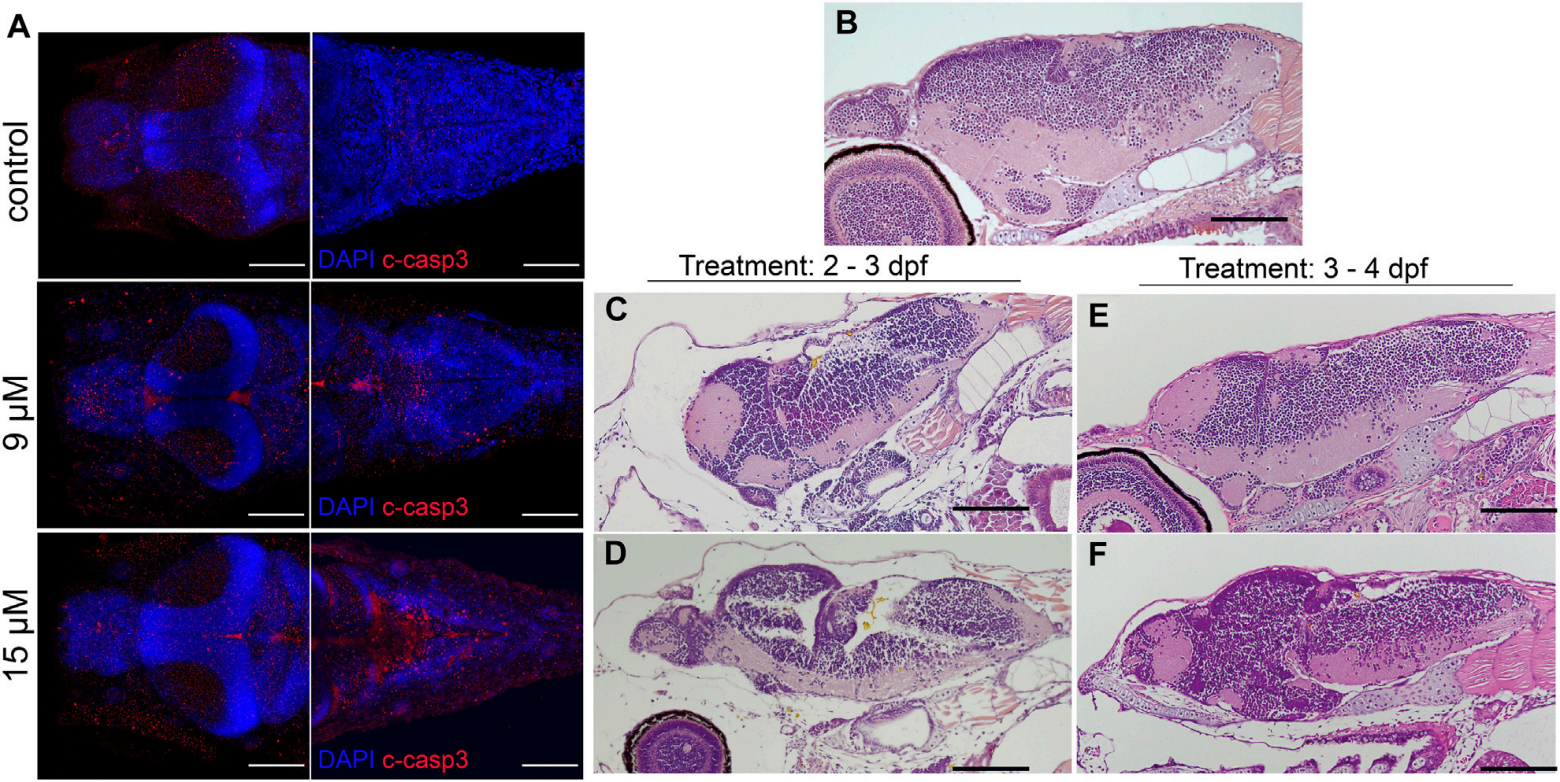
**Bilirubin-induced neurological damage (BIND) is detected in the larval brain:** **(A)** Apoptotic cells were detected in the brains of control and hyperbilirubinemia larvae at 4 dpf. DAPI (blue) highlights the brain structure, and cleaved caspase 3 (C-Casp3) (red) labels apoptotic cells. Dorsal views of fore brain (left) and hind brain (right) are shown for control, 9 μM and 15 µM bilirubin treatment groups. **(B–F)** H&E-stained paraffin sections of 6 dpf larval brains. **(A)** Intact and healthy brain tissue in control larva; deformed brain tissue in larvae that received **(B)** 9 µM or **(C)** 15 µM bilirubin between 2**–**3 dpf. **(D)** Larva that received 9 µM bilirubin between 3 - 4 dpf had normal brain tissue. **(E)** Larva that was exposed to 15 µM bilirubin between 3**–**4 dpf had slight deformation in brain tissue. Scale bars: 100 µm.

An increase in apoptosis was observed in larvae treated with 15 µM bilirubin (Figure 6A). Degeneration in the brain tissue was visible on tissue sections of larvae, several days after washout, at 6 dpf (Figures 6B–F). Bilirubin accumulation in the brain led to edema and shrinkage of the neuronal tissue in 9 µM-exposed larvae (Figure 6C). Major acellular areas and fragmentation of the tissue were detected in the forebrain and hindbrain of 15 µM-exposed larvae (Figure 6D). These defects were less pronounced in the group that received bilirubin between 3 and 4 dpf (Figures 6E,F). Otic vesicles were deformed in all groups regardless of the exposure time (Supplementary Figure S6).

### 3.5 Larval neurological functions are impaired in hyperbilirubinemia

**FIGURE 7:**
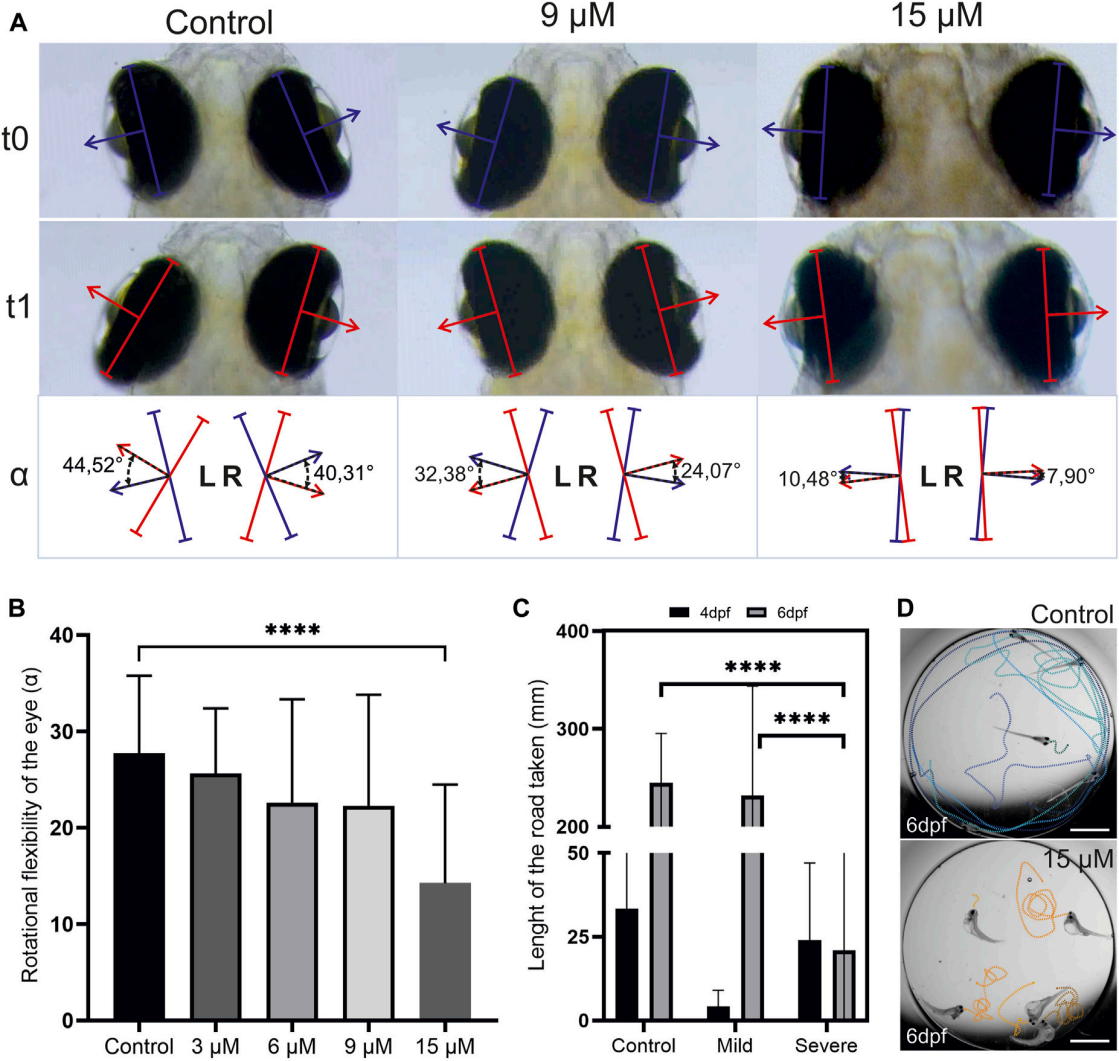
**Hyperbilirubinemia affects eye reflexes and swim behavior:** **(A)** Turn of the eyes in response to light stimulation is displayed. Turn of the eye between t_0_ and t_1_ was measured as turn angle alpha. **(B)** Average turn angle of the eyes of larvae exposed to 3, 6, 9 and 15 µM doses of bilirubin is displayed. **(C)** Average swim distance of controls and larvae exposed to 9 µM bilirubin between 2 and 3 dpf at 4 and 6 dpf is displayed. Data are the mean ± SEM. (A″-C″) n = 20, 18, 22, 20, and 22 for control, 3, 6, 9, and 15 µM. **(D)** Directional swim behavior of the control and swirling swim behavior of the severely affected larvae are displayed.

In severe cases of hyperbilirubinemia, BIND may present as paralyzed or limited gaze or weakness of eye muscles (Rose and Vassar, 2015; Magai et al., 2020). Eye reflexes of larvae with hyperbilirubinemia were measured at 6 dpf (2 days after washout). To this end, the turn angle of the eyes in response to a light stimulus in a semidark environment was measured, and the average turn angle was quantified (Figures 7A,B). This experiment demonstrated that the turn angle of the eyes decreased in a dose-dependent manner.

Motor dysfunction caused by BIND includes dystonia (increased or decreased muscle tone) and excessive abnormal movements such as writhing movements (Le Pichon et al., 2017). The swimming behavior of the hyperbilirubinemic larvae with mild and severe phenotypes was recorded at 4 dpf and 6 dpf to test acute and lasting effects. Larvae with the mild phenotype initially displayed hypoactivity, swimming much less than controls at 4 dpf. However, at 6 dpf, their activity levels increased and matched those of the control group (Figure 7C). Larvae with a severe phenotype had a curved body shape, and they were not able to swim directionally but displayed a swirling type of swimming at both 4 and 6 dpf (Figure 7D). Compared to the control, these larvae were observed to display more activity and irritability (not quantified). At 6 dpf, these severely affected larvae exhibited activity levels far below both the control group and larvae with a mild phenotype (Figure 7C).

## 4 Discussion

Although rodent models of hyperbilirubinemia and bilirubin-induced neurological damage (BIND) have been developed before, alternative models are still needed to recapitulate newborn hyperbilirubinemia to the full extent (Amini et al., 2021). Here, the first zebrafish hyperbilirubinemia model is generated via exposure of early zebrafish larvae to external bilirubin. To mimic the human condition, the maturation of organs and expression of bilirubin metabolism enzymes during the first 5 days of zebrafish development were considered carefully. Since liver maturation is completed by day 5 and the gut gains functionality by day 4, the period between 2–4 dpf was considered ideal for neonatal hyperbilirubinemia development (Wallace et al., 2005; Chu and Sadler, 2009). One limitation of the study was the insolubility of bilirubin in embryo water, which was overcome by the use of a BSA carrier. After testing exposure between 2–3 dpf and 2–4 dpf, it was found that a 24-h exposure between 2–3 dpf with 9 or 15 µM bilirubin induced a condition comparable to severe newborn hyperbilirubinemia. 15 μM caused a more severe phenotype, and most larvae were highly damaged or dead by 5 dpf. However, 9 µM bilirubin caused a strong phenotype but better survival of the exposed larvae. Finally, 48-h exposure caused stronger phenotypes at all concentrations, leading to lethality in 15 µM-treated larvae and a severe phenotype in a small fraction of 6 µM-exposed larvae. In summary, dose- and time-dependent bilirubin accumulation and induction of a severe phenotype were observed.

Shifting the exposure onset time by 1 day induced a milder phenotype in overall larvae, suggesting that tolerance to bilirubin increases at 3 dpf. The tissues involved in bilirubin metabolism are the liver, kidneys and gut in humans, but whether this is conserved in zebrafish was not studied previously (Fujiwara et al., 2018). Here, tissue sections of bilirubin-treated larvae of all groups were examined to check for bilirubin presence and any bilirubin-induced damage. The fish that received bilirubin between 3–4 dpf had better morphology as well as healthier liver and gut tissue at 6 dpf. The presence of bilirubin in these tissues 2 days after the washout suggests that the bilirubin that accumulated in the body is processed by the liver and gut without damaging these tissues. On the other hand, fish that received bilirubin between 2–3 dpf had damaged liver and gut, although bilirubin was detected in these tissues as well. Damage to the liver and gut may be related to interference with the development of these tissues, which is likely to hinder bilirubin clearance. This finding is important to show the conservation of bilirubin clearance mechanisms between humans and zebrafish.

Total bilirubin retained in the larval body was quantified with a spectrophotometric method, and the amount of bilirubin in the body increased in a dose-dependent manner, in agreement with the phenotypic observations. Bilirubin accumulation in the brain was compared among groups that received different doses, and relative bilirubin accumulation in the brain reached a maximum in groups treated with 9 µM for 48 h or 15 µM for 24 h. The quantitation confirmed that 6 µM bilirubin resulted in less accumulation in the body and brain compared to 9 and 15 μM, which are doses that cannot be tolerated by 2–3 dpf zebrafish. This threshold of 9 µM can be considered similar to human significant hyperbilirubinemia with total serum bilirubin (TSB) ≥ 12 mg/dL (Olusanya et al., 2018). In humans, total serum bilirubin levels as well as the age of the infant are important indications for the risk of BIND or KSD development; however, these factors are not completely predictive. The reasons leading to individual variance in susceptibility to developing BIND or KSD are not fully understood. The zebrafish model exhibited a similar variance leading to the development of mild or severe phenotypes in equally treated and age-matched zebrafish larvae, which can provide a means for studying the underlying mechanisms.

Next, BIND and related symptoms were investigated in the developed zebrafish model. Bilirubin accumulation in the brain and otic vesicles in a dose- and time-dependent manner was demonstrated. Bilirubin accumulated in the telencephalon and rhombencephalon (cerebellum) in 9 µM-exposed larvae, while increasing the dose to 15 µM resulted in intense accumulation in the optic tectum (mesencephalon). Similarly, in infants with bilirubin encephalopathy, the hippocampus and ocular basal ganglia brain regions are affected (Wisnowski et al., 2016). The brain compartments telencephalon, optic tectum, and cerebellum shrank, and edema in the brain and eye sclera caused ballooning when toxic 9–15 µM doses were applied. The eye tissue was smaller, indicating a delay in development, and the sclera width was increased. BIND in the zebrafish model was further confirmed by histopathology, and degeneration of the brain tissue was shown. An increase in apoptosis in the brain was also demonstrated with cleaved caspase 3 staining. When considered together, these findings indicate that the zebrafish model can be used to induce BIND when 9–15 µM bilirubin is applied between 2–3 dpf.

Functional tests were performed to compare zebrafish phenotypes to human acute and chronic bilirubin encephalopathy (ABE and CBE). Curved body and deteriorated muscles as well as shorter body in zebrafish with severe phenotype resembles opisthotonos that results from dramatic contraction of body muscles in severely affected infants. Problems with posture are generally accompanied by problems in movement control in affected infants. When swimming behavior was analyzed, it was found that these severely affected larvae responded to touch but were not able to swim directionally and displayed a swirling type of motion. These larvae had problems terminating motion, but the swim distance was not increased due to the swirling swim style. On the other hand, larvae with a mild phenotype had normal body posture and could perform directional swimming at 6 dpf in a comparable manner to the control group. Interestingly, in the acute period at 4 dpf, these larvae displayed decreased activity and lethargy. This phenotype is comparable to the sleep tendency and increased lethargy observed in BIND patients (Magai et al., 2020). Upward gaze palsy is another symptom of bilirubin encephalopathy, reproduced in the zebrafish model as an eye movement restriction phenotype (Usman et al., 2018). The turn angle of the eyes upon light stimulation decreased in the 9 µM-treated group and severely dropped in the 15 µM-treated group. It should be noted that scleral widening also increased in the 15 µM-treated group, which may be the main reason for gaze limitation.

## 5 Conclusion

The zebrafish newborn hyperbilirubinemia model reported here showed that bilirubin exposure before development of the liver induces toxicity in a dose-dependent manner. Larvae with hyperbilirubinemia were proven to be a good model for BIND. Not only does bilirubin accumulate in neural tissues, but it also causes damage to the brain and eyes and induces lethargy. Strikingly, recovery of larvae from hyperbilirubinemia after washout was incomplete, and even the mild cases (treated with 9 µM bilirubin) had defects in liver and brain tissues as well as some limitation of eye rotation at 6 dpf. Individual variability in susceptibility to bilirubin was observed, which can provide a basis for mechanistic studies. The model provides an alternative *in vivo* model for studying BIND mechanisms and prevention or treatment strategies.

## Data Availability

The datasets presented in this study can be found in online repositories. The names of the repository/repositories and accession number(s) can be found in the article/Supplementary Material.
