# Supplementary material for: A zebrafish model for studying the mechanisms of newborn hyperbilirubinemia and bilirubin-induced neurological damage
Source: Front Cell Dev Biol. 2023 Nov 14;11:1275414. doi: 10.3389/fcell.2023.1275414 (PMC10682072; doi:10.3389/fcell.2023.1275414)
Supplement: Supplementary file 1 [file DataSheet1.docx]

**SUPPLEMENTARY INFORMATION**

**A zebrafish model for studying the mechanisms of newborn hyperbilirubinemia and bilirubin-induced neurological damage**

Metehan Guzelkaya^1, #^, Ebru Onal^1, 2, #^, Emine Gelinci^1^, Abdullah Kumral^3^, Gulcin Cakan-Akdogan^1, 4, *^

**
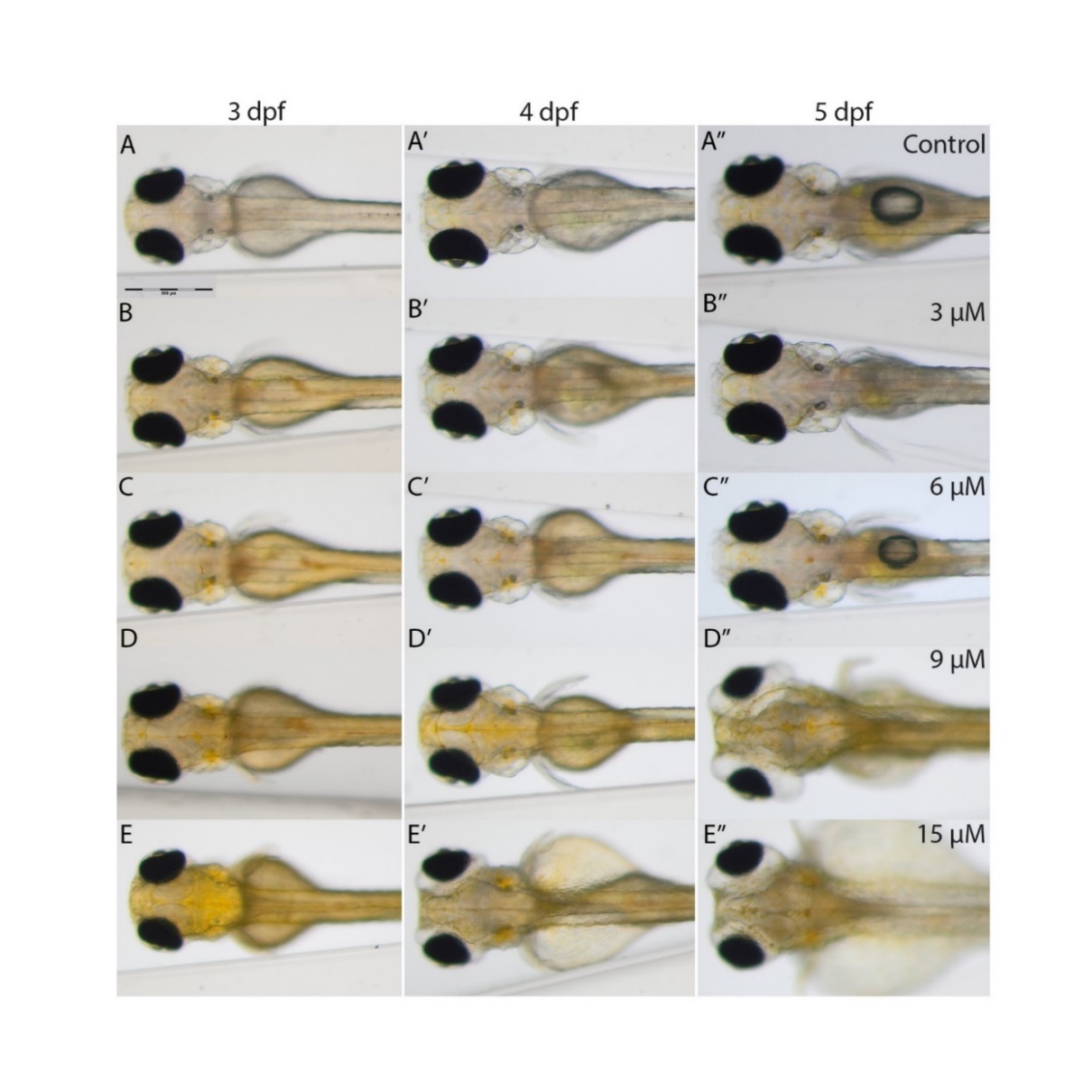
**

**Fig. S1.** **Dorsal Images of bilirubin exposed larvae.** Dorsal images of larvae that were exposed to: **A-A’’**) carrier, **B-B’’**) 3 µM, **C-C’’**) 6 µM, **D-D’’**) 9 µM, and **E-E’’**) 15 µM bilirubin between 2 - 3 dpf. Representative images recorded at the end of treatment (3 dpf, left column), 1 day after washout (4 dpf, middle column) and 2 days after washout at 5 dpf (right column). Scale bar: 200 µm, n=40


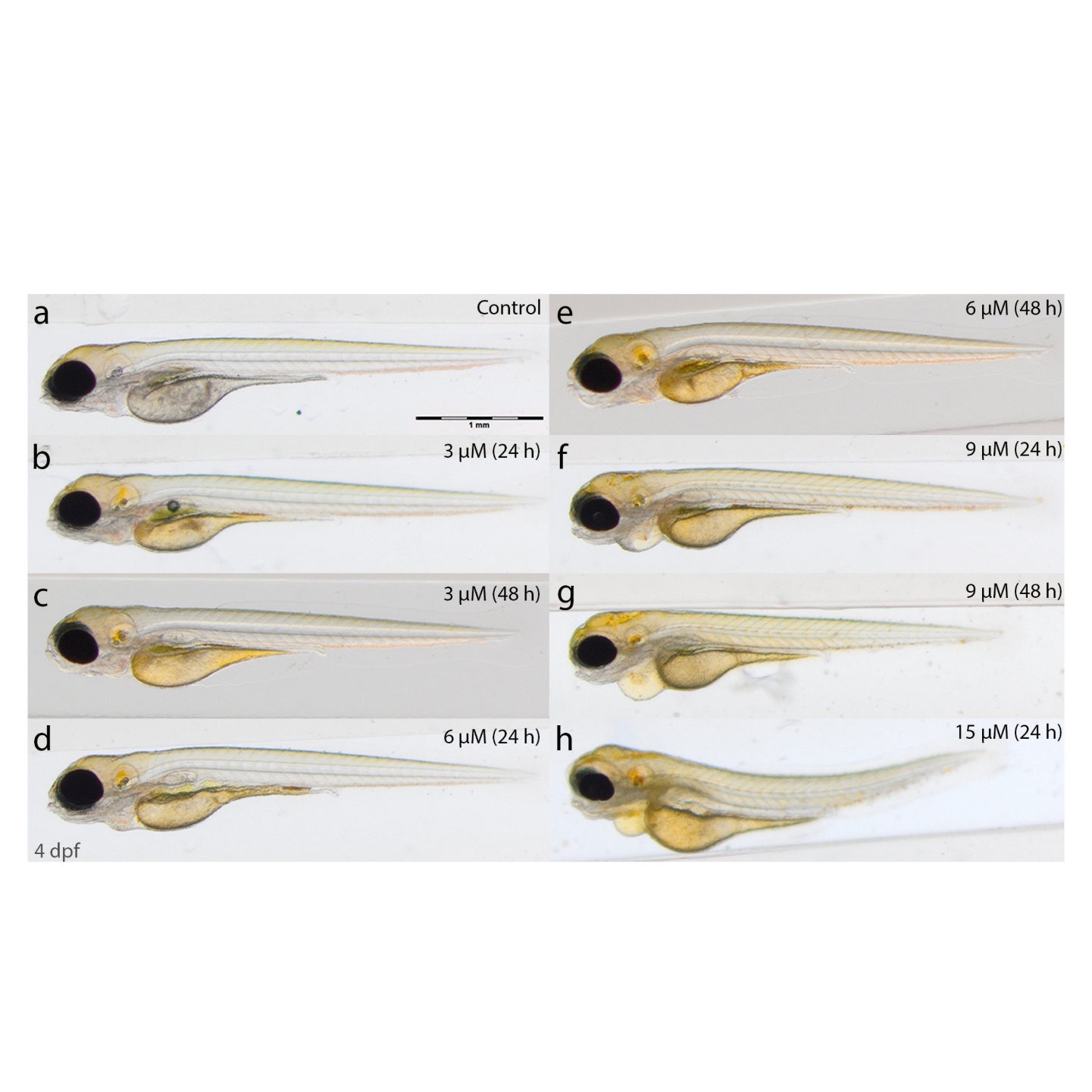


**Fig. S2. Representation of the entire body of Casper larvae at 4dpf.** Visualization of Casper larvae decreasing body length based on bright field images at 4 dpf, gradually increasing bilirubin doses to 3, 6, 9 (24h and 48h) and 15 µM. Scale bar: 200 µm.


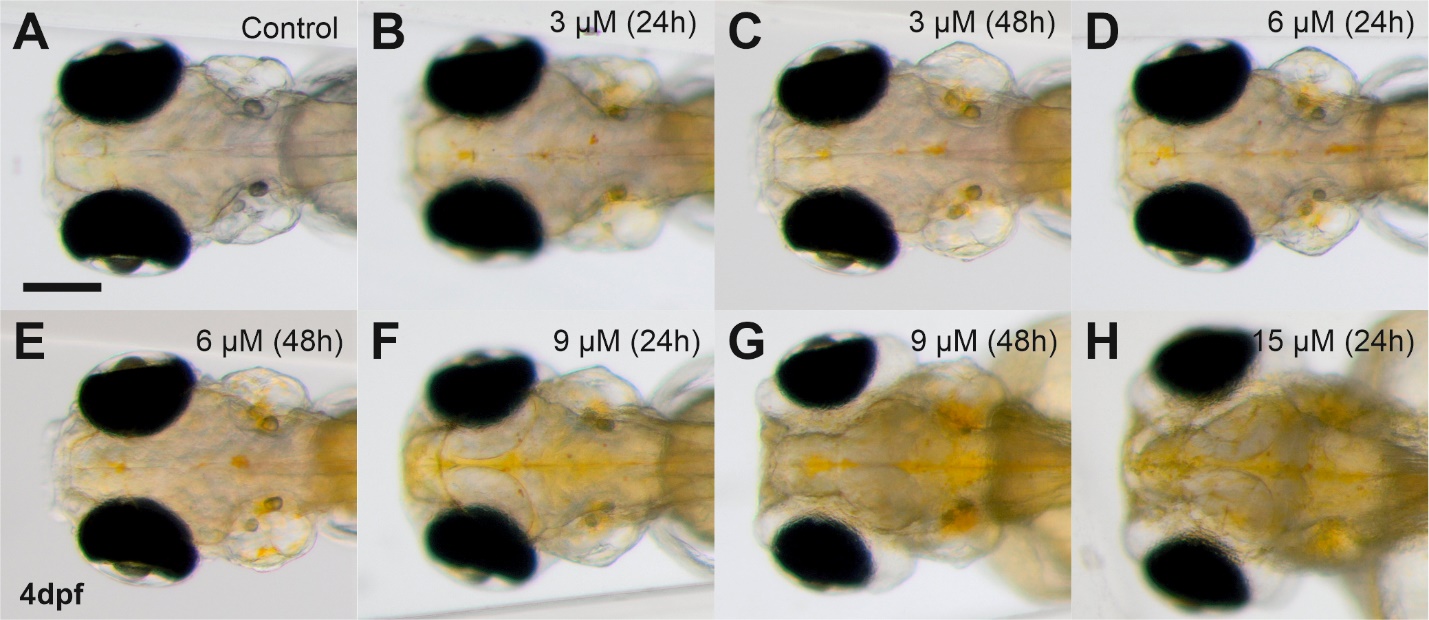


**Fig. S3. Dorsal head images of zebrafish larvae treated with bilirubin.** Casper larvae treated with bilirubin for 24 h or 48 h with 3 – 15 µM bilirubin were imaged at 4 dpf. Doses and treatment durations are indicated on each image. Scale bar: 200 µm.


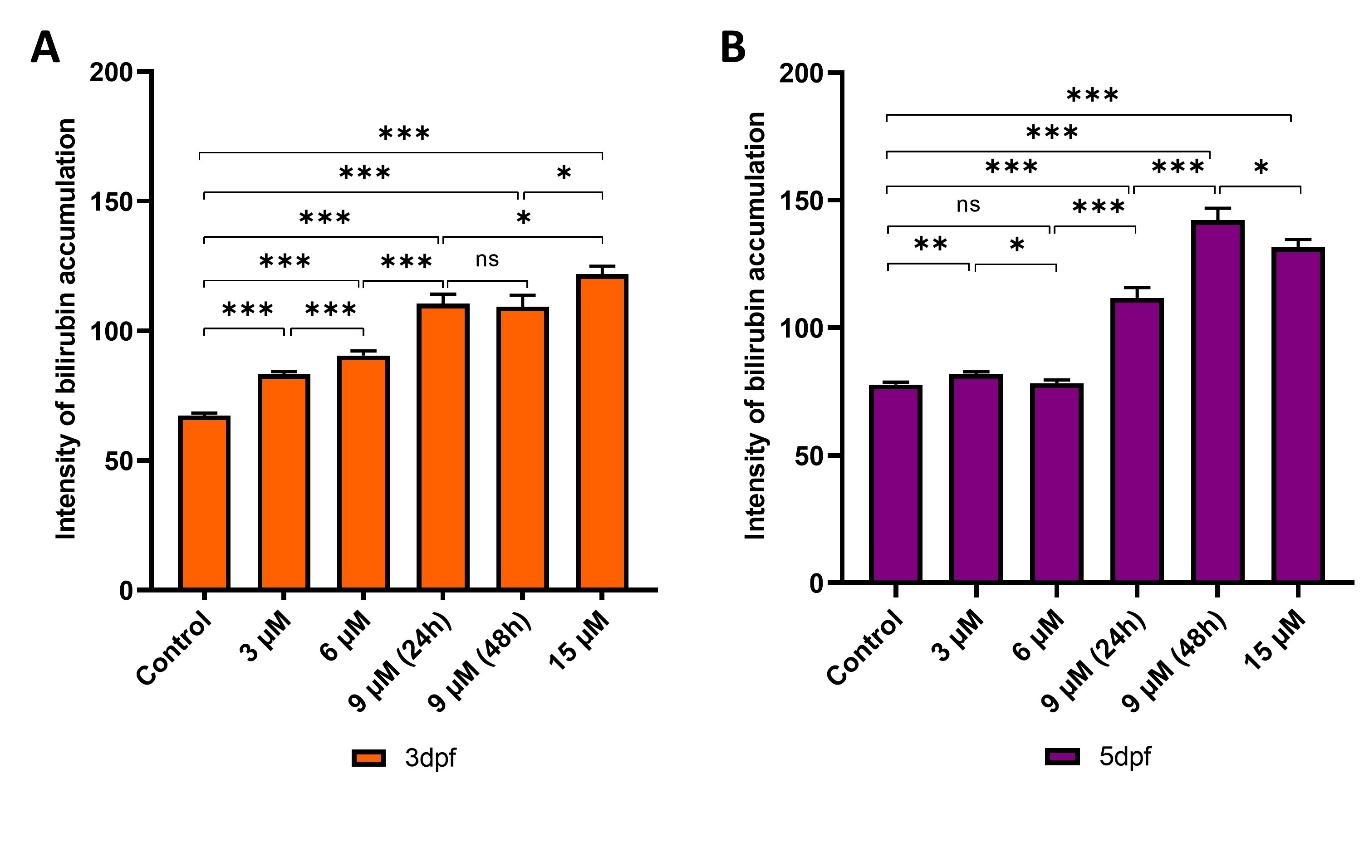


**Fig. S4.** **Analysis of bilirubin accumulation in neural tissues at 3 and 5 dpf.** Quantification of mean gray values of bilirubin accumulation in the middle part of the brain at **A)** 3 dpf and **B)** 5 dpf. (n=20, 15, 12, 11, 11 and 11 at 3 dpf ; n=18, 14, 32, 11, and 11 at 5 dpf for control, 3, 6, 9 (24h and 48h) and 15µM). *P<0.05; **P<0.01; ****P<0.0001; NS, not significant (one-tailed t-test).


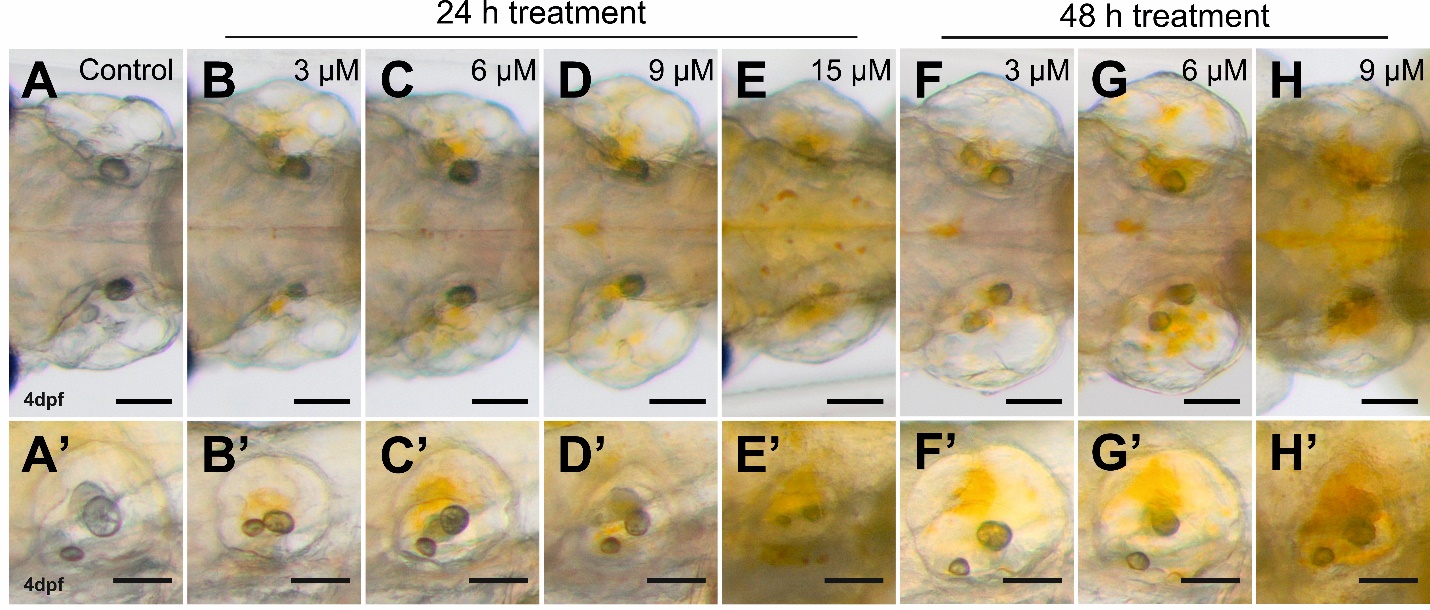


**Fig. S5.** B**ilirubin accumulation in otic vesicles: A-H)** Dorsal view of the head, **A’-H’)** lateral view of the otic vesicle showing the bilirubin accumulation in otic vesicles. Scale bar 50 µm.


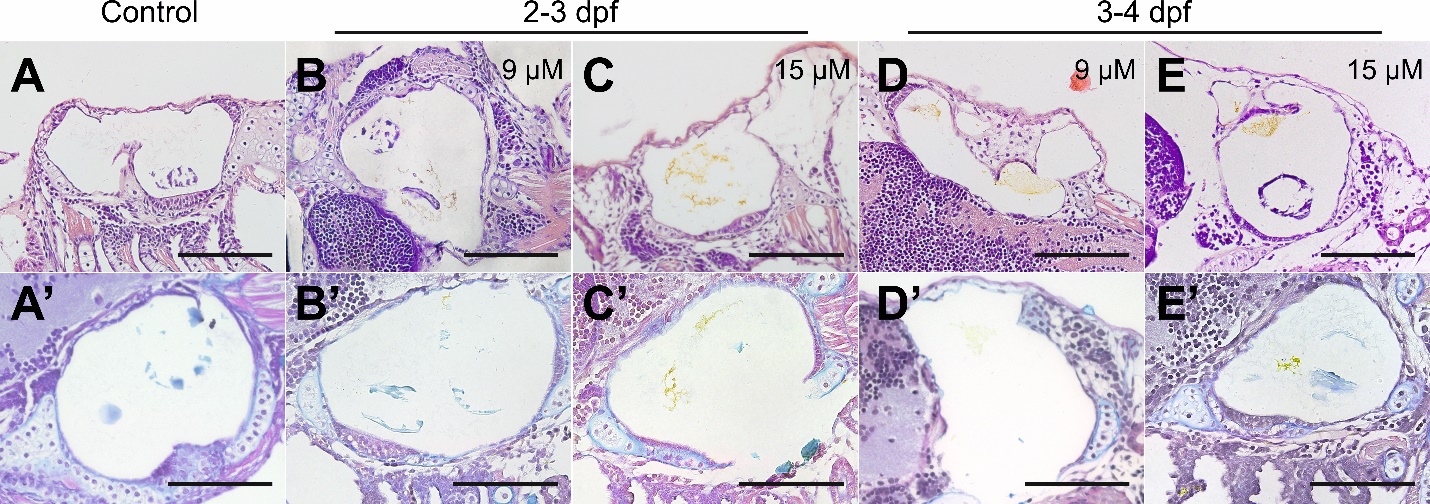


**Fig. S6 Histopathology of otic vesicles after bilirubin exposure: A-E)** H & E staining of otic vesicle sections. **A’-E’)** Masson-Trichrome staining of otic vesicle sections. Scale bar 50 µm.
